# Benchmarking Feature Selection Methods and Prediction Models for Flowering Time Prediction in Maize

**DOI:** 10.3390/ijms27041635

**Published:** 2026-02-07

**Authors:** Yan Du, Nianhua Jia, Yueli Wang, Ronglan Li, Ying Lu, Tobias Würschum, Xintian Zhu, Wenxin Liu

**Affiliations:** 1 State Key Laboratory of Maize Bio-Breeding, National Maize Improvement Center, College of Agronomy and Biotechnology, China Agricultural University, Beijing 100193, China; 2 Sanya Institute of China Agricultural University, China Agricultural University, Sanya 572025, China; 3 Institute of Plant Breeding, Seed Science and Population Genetics, University of Hohenheim, 70599 Stuttgart, Germany

**Keywords:** genomic prediction, machine learning, flowering time, feature selection, SHAP

## Abstract

Flowering time is a fundamental trait that determines crop adaptation and yield stability. To accurately predict flowering time and identify key regulatory factors, it is necessary to extract biologically meaningful signals from high-dimensional and multi-omics datasets. Although machine learning has been increasingly applied in plant genomics, there is still limited research on how feature selection (FS) methods and genomic prediction (GP) models affect the prediction of flowering time and gene discovery, particularly regarding the combination of different FS and GP approaches and the interpretability of prediction models. To address this gap, we conducted a large-scale benchmarking study that jointly evaluated seven feature selection methods and six prediction models, resulting in 42 FS–GP combinations. By integrating SNP and transcriptomic data, we assessed predictive performance and further interpreted model outputs using SHAP (SHapley Additive exPlanations) within a random forest (RF) framework to quantify feature contributions. This strategy successfully identified known flowering time regulators in maize, including *ZmMADS69* and *ZmRap2.7*, and revealed additional candidate genes potentially involved in the flowering regulatory network. Overall, this study offers valuable insights into the genetic regulation of flowering time in maize and provides an effective framework for discovering candidate genes from multi-omics data for crop improvement.

## 1. Introduction

Maize (*Zea mays* L.) is a globally important staple crop with considerable economic value in feed, food, industrial production, and energy sectors [1,2]. It is estimated that by 2050, the global population will reach 9.5 billion. Increasing the yield per unit area and breeding improved varieties in a short period of time have become essential strategies to address food security challenges posed by population growth [3]. However, achieving these breeding goals has become increasingly challenging due to the impact of extreme climatic events [4]. Among the agronomic traits targeted in maize breeding, flowering time is a typical complex quantitative trait controlled by many loci and strongly influenced by environmental conditions. Flowering time represents a critical developmental stage in the plants’ lifecycle, playing a decisive role in reproduction and seed yield. And flowering time is considered a key selection criterion in maize breeding as it reflects the adaptability of varieties to local environmental conditions [5,6,7]. In recent years, methods such as population analysis, comparative genomics, and mutant screening have contributed to the growing number of cloned flowering time genes in maize [8,9,10]. However, research on maize flowering time still faces numerous challenges, and the discovery of new regulatory loci remains essential.

Genomic prediction (GP) has been widely recognized as a powerful tool in plant and animal breeding. It enables the prediction of individual phenotypes or complex quantitative traits through the integration of genome-wide marker information. By integrating genome-wide markers with the phenotypic data of a training population, the breeding values of candidate lines can be accurately estimated without phenotyping, whereby selection intensity can be increased, breeding costs reduced, and the process of genetic improvement accelerated [11,12,13].

With the widespread adoption of sequencing technologies, the number of markers has increased exponentially, but high-dimensional data are confronted with the “curse of dimensionality,” high collinearity, noise accumulation, and model overfitting, which also substantially increases computational cost and model training time [14]. Therefore, the identification of key features contributing most to trait variation has become increasingly important. Feature selection (FS), by removing irrelevant, redundant, or noisy features, can significantly enhance model robustness and predictive performance. While various methods for dimensionality reduction and model improvement have been systematically evaluated, several limitations remain. Most evaluations have focused on human or animal datasets, with limited validation in crop systems [15,16]. Additionally, only a limited number of feature selection methods and prediction models have been compared, making it difficult to comprehensively evaluate different strategies [16]. Furthermore, input data were typically restricted to SNP markers, with limited incorporation of transcriptomic information, thereby failing to reflect the complexity of future breeding data environments [17]. Some studies have performed feature selection and model training on the full dataset simultaneously, neglecting the strict separation of training and test sets, which may lead to overly optimistic performance assessments and reduce reliability [15]. Lastly, many methods still face challenges regarding model interpretability, limiting their practical utility in breeding decisions.

SHAP (SHapley Additive exPlanations), a widely applied explainable AI approach, has been employed to interpret black-box machine learning models [18]. Unlike traditional feature importance analyses that only provide global rankings, SHAP allows the contribution of individual features (e.g., markers or expressed genes) to be quantified for each sample. This sample-level interpretability is particularly valuable for genetically heterogeneous plant populations, where the effects of specific loci may vary substantially across individuals. This enables the understanding of not only which genes are important but also how they influence specific phenotype predictions, enhancing both model interpretability and biological relevance [19]. Although its use in large-scale plant omics data remains limited, SHAP has been successfully applied in multiple fields, highlighting its potential for crop complex trait prediction and gene identification [20].

Flowering time is a classic quantitative trait in crops, with a genetic basis comprised multiple regulatory interactions and a complex genetic architecture. Despite the increasing application of machine learning techniques in bioinformatics, systematic research on the prediction of flowering time-related genes remains limited, particularly for the integration of feature selection and model building strategies, where significant theoretical and methodological gaps remain. This study focuses on flowering time in maize and systematically compares the performance of different feature selection methods to fill this gap by providing a large-scale benchmark evaluation of feature selection strategies. Seven feature selection strategies and six prediction models are systematically applied to SNP and transcriptomic data. SHAP values were further used to interpret known functional genes and to identify novel candidate genes. The findings provide guidance for selecting optimal methodologies and contribute to a deeper understanding of the genetic basis of complex traits.

## 2. Results

### 2.1. Pipeline of Feature Selection Methods and Prediction Models for Complex Trait Prediction

**Figure 1:**
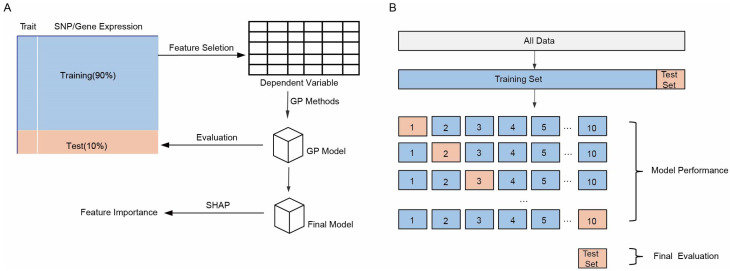
Schematic presentation of the framework for complex trait prediction. (**A**) Workflow of the prediction pipeline. (**B**) Schematic diagram of the ten-fold cross-validation principle for genomic prediction (GP) model performance. Blue represents the training set, and orange represents the test set.

To systematically explore the combined effects of feature selection methods and prediction model on the predictive performance, we developed a robust analytical framework that integrates data processing, feature selection, model evaluation and model explanation (Figure 1A). Special attention was given to the stability of feature selection strategies in high-dimensional omics data, along with the potential benefits of integrating SNP and transcriptome information for improving prediction accuracy. In this high dimensional setting, the performance of integrated data was examined in depth to establish a more robust approach for complex trait modeling. First, a ten-fold cross-validation scheme was applied to the dataset to assess model performance, that was, 90% of the data was used as the training set, and 10% of the data was used as the test set in each iteration of model performance (outer loop). Within each run of model performance assessment, feature selection was performed in the training set with nine-fold cross-validation (inner loop) to identify the most important features recognized according to each method, and the selected features were then used to train the respective prediction models. The trained models were subsequently evaluated with the test set (Figure S1). It is important to emphasize that feature selection and model evaluation are two independent steps. Therefore, the evaluation of model performance must be based on data not used for training to ensure the objectivity and reliability of the results. In addition, the SHAP value framework was applied to interpret the models and reveal the contribution of individual features to the prediction outcome.

During this process, the predictive performance of each model was systematically assessed using ten-fold cross-validation (Figure 1B). For this, the entire dataset was partitioned into ten mutually exclusive subsets and in each iteration, nine subsets were used for model training and feature selection, while the remaining subset was used for testing. This strategy effectively reduces randomness caused by data splitting and enhances the robustness and reliability of model performance evaluation. The results obtained from the ten-fold cross-validation provide a solid foundation for subsequent model comparison and feature importance analysis.

### 2.2. The Performance of Linear and Nonlinear Feature Selection on Flowering Time Prediction

**Figure 2:**
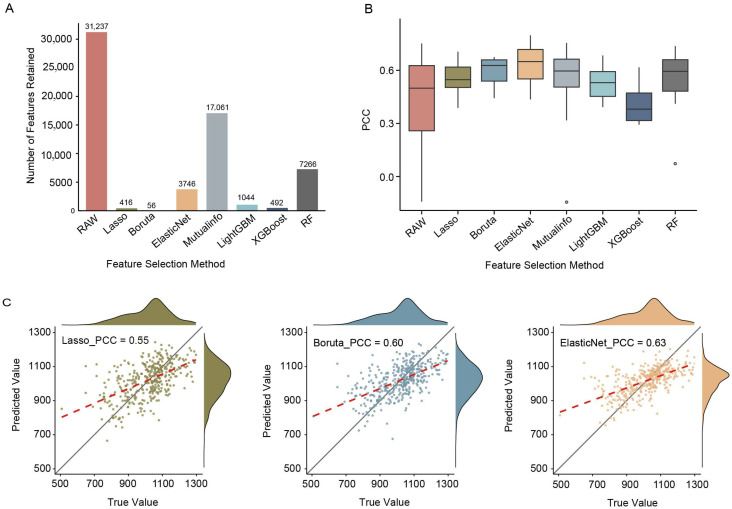
Comparison of rrBLUP across different feature selection methods. (**A**) The average number of features retained by each feature selection method. (**B**) The box plot shows the distribution of Pearson correlation coefficient (PCC) across different feature selection methods. (**C**) Scatter plots comparing predicted versus true trait values for selected models. Each subplot includes a regression line (red dashed) and a diagonal reference line, illustrating the consistency between prediction and observation. RF, random forest.

Feature selection can greatly reduce computational and resource costs. But which feature selection methods are capable of identifying the most relevant features without compromising prediction accuracy? This remains a key question that underlies this study. To address this, we integrated multiple feature selection strategies and subsequently examined their practical contributions to prediction model accuracy. Specifically, we used flowering time data from 388 maize inbred lines (Table S1) as the target phenotype and leveraged seedling-stage transcriptomic profiles (Table S2) as the feature matrix for prediction. Based on this dataset, we conducted a systematic evaluation of seven representative feature selection strategies. The feature selection methods included both linear approaches (Lasso and ElasticNet) and nonlinear ones (mutual information, RF, Boruta, LightGBM and XGBoost). After feature selection, the number of features was reduced from the original 31,237 to 56–17,061, corresponding to a compression ratio of approximately 45.0–99.8%. Among all methods, Boruta achieved the most substantial reduction, retaining only 56 features, followed by Lasso with 416 features, whereas mutual information preserved the largest number of features (17,061). These results highlight the substantial differences in the feature selection approaches to remove redundancy and distill informative signals (Figure 2A).

Our results showed that compared with the full set of features (RAW), all feature selection strategies except XGBoost led to performance gains when combined with rrBLUP as prediction model (Figure 2B). Among the linear approaches, ElasticNet, which integrates both L1 and L2 regularization, demonstrated superior capability in retaining relevant features and enhancing prediction stability compared to Lasso. However, among the nonlinear methods, tree-based models such as LightGBM and XGBoost exhibited notable performance differences, as LightGBM significantly outperformed XGBoost. Interestingly, features selected by RF showed robust compatibility, suggesting that some tree-based methods may still yield variables suitable for linear models.

All feature selection methods exhibited a clear linear relationship between the predicted and observed phenotypic values under the rrBLUP model, and the overall trend of the regression lines indicated a strong linear fitting ability. Among them, the ElasticNet feature selection method performed the best, achieving the highest mean Pearson correlation coefficient (PCC = 0.63 ± 0.13), while retaining an average of 3746 features. Its predicted values aligned most closely with the fitted trend line and showed the fewest outliers, demonstrating excellent predictive accuracy and stability (Figure 2C). These results suggest that appropriate feature filtering not only enables effective dimensionality reduction but also retains the key predictive signals.

### 2.3. Machine Learning Models Achieve Higher Accuracy for Flowering Time Prediction

**Figure 3:**
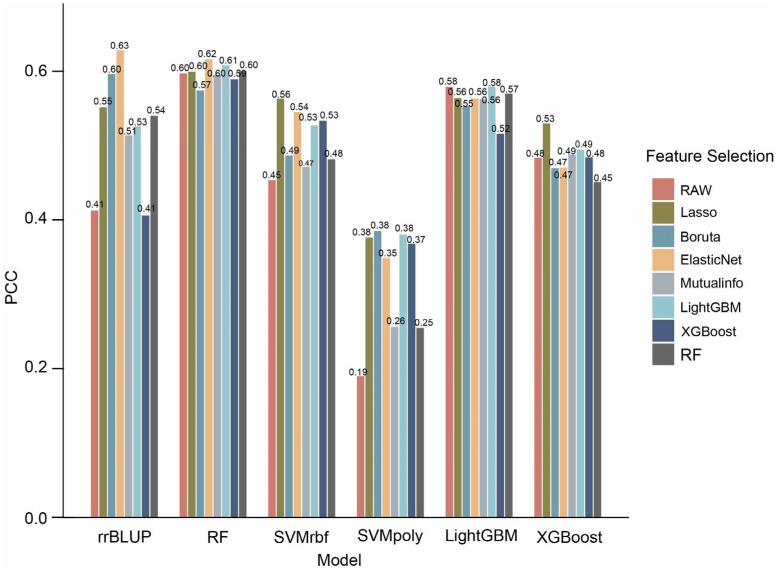
Assessment of model performance in predicting flowering time from transcriptome-based features. Comparison of prediction performance by Pearson correlation coefficient (PCC) for combinations of seven feature selection methods and five machine learning models.

To systematically investigate the effectiveness of various combinations of feature selection method and prediction model, we conducted a comprehensive comparison to evaluate their impact on prediction performance. By assessing each combination, we aimed to uncover the interactions between feature selection strategy and prediction model and provide insights for selecting the optimal pairing. We evaluated five machine learning prediction models, RF, SVMrbf, SVMpoly, LightGBM, and XGBoost, combined with seven feature selection methods. Across different prediction models, predictions based on the full set of raw features consistently outperformed rrBLUP (except for SVMpoly), indicating that machine learning algorithms are better suited to capture complex nonlinear relationships than traditional linear models (Figure 3, Table S3). After applying feature selection for dimensionality reduction, most models exhibited improved predictive ability, most notably rrBLUP, highlighting the value of feature selection in removing irrelevant features and mitigating the “curse of dimensionality”. As a result, feature selection enables certain models to more effectively capture trait associated signals.

Among all combinations, the RF prediction model consistently demonstrated the best overall performance, followed closely by LightGBM. Notably, the ElasticNet-rrBLUP combination achieved the highest prediction accuracy (PCC = 0.63 ± 0.13), with ElasticNet-RF ranking second (PCC = 0.62 ± 0.09). Surprisingly, although Boruta retained an average of only 56 features, more than half of the tested prediction models combined with Boruta achieved higher prediction accuracy compared with using the full set of features. This suggests that Boruta selects highly informative and remarkably stable features. In contrast, combinations involving SVMpoly as prediction model generally performed the worst, and even the raw feature set failed to enable SVMpoly to capture the key predictive signals. These results underscore the importance of choosing an appropriate genomic prediction model to optimize prediction accuracy.

### 2.4. Performance Evaluation of Omics Data Integration for Flowering Time Prediction

**Figure 4:**
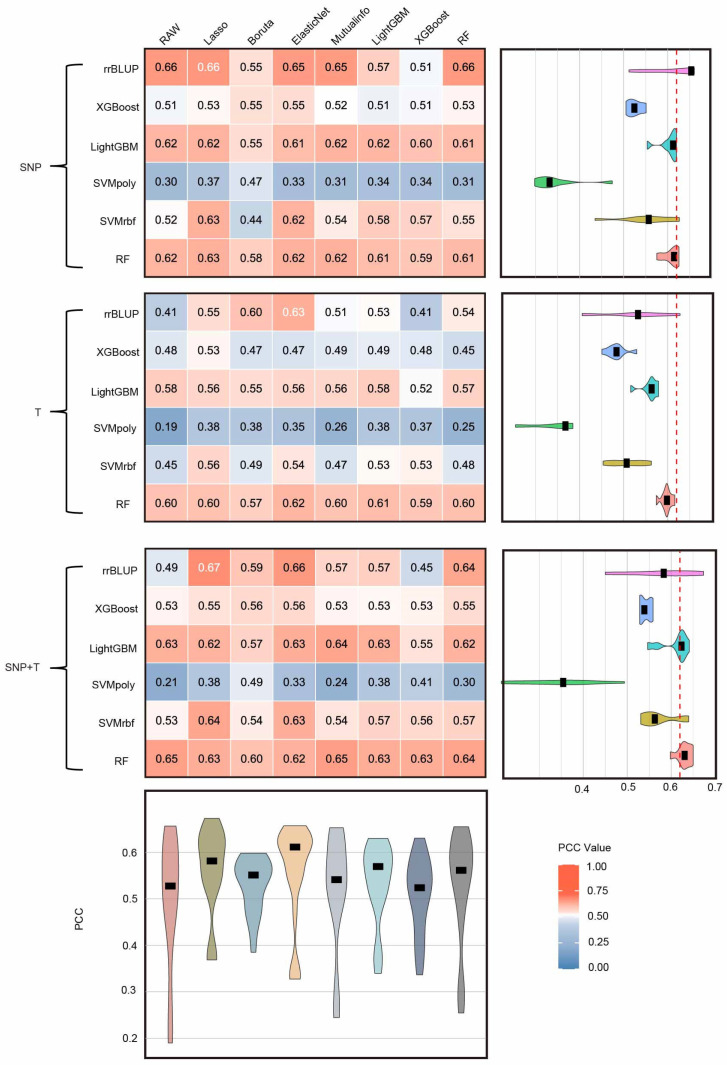
Performance evaluation of different feature selection methods and machine learning models across three data types (SNP, Transcriptome, and the combined SNP + Transcriptome). The heatmap on the left represents the prediction accuracy (Pearson correlation coefficient, PCC values) of different feature selection and prediction model combinations, in which, the white number means the top prediction performance among the whole FS-GP combinations in each data type. The violin plots on the right depict the PCC value distribution for each model, with the red dashed line indicating the PCC = 0.62. The violin plot at the bottom compares the PCC distributions across all model feature selection combinations. T represents transcriptome.

Studies have shown that machine learning algorithms have a significant impact on prediction using different omics data types [21]. Here, we selected three datasets for comparative analysis: SNP data, transcriptomic data, and their combination. The results revealed that integrative multi-omics modeling showed similar performance to single-omics approaches. With individual omics data, SNP data showed overall higher predictive power than transcriptomic data, with rrBLUP, RF, and LightGBM achieving the best performance. In contrast, models based solely on transcriptomic features produced slightly lower correlations between predicted and true values, suggesting that transcriptome signals alone capture less of the phenotypic variance. When the two data types were combined, the SNP + T integration only increased the average Pearson correlation coefficient to 0.67, representing improvements of approximately 1.52% over SNP alone (Figure 4 and Figure S2).

Among the feature selection strategies, both Lasso and ElasticNet exhibited superior overall performance. They maintained relatively high PCC values, with median PCCs of 0.58 (IQR: 0.54–0.63) and 0.62 (IQR: 0.54–0.63), respectively, and ranked among the top performers in more than 70% of model feature selection combinations. Boruta achieved moderate overall performance (median PCC = 0.55, IQR: 0.49–0.57), but demonstrated strong compatibility with the RF model, significantly reducing the number of features while still retaining key information for prediction. Based on these findings, we propose the following optimization strategy: Prioritize Lasso, ElasticNet or Boruta for dimensionality reduction in high-dimensional multi-omics data to greatly reduce the number of features while retaining as much informative signals as possible, then employ RF as the primary prediction model to fully leverage its robustness and interpretability in handling high-dimensional, heterogeneous data, enabling efficient, accurate, and reliable phenotype prediction (Table S4).

### 2.5. Potential to Predict Flowering Time Genes Through SHAP Values

**Figure 5:**
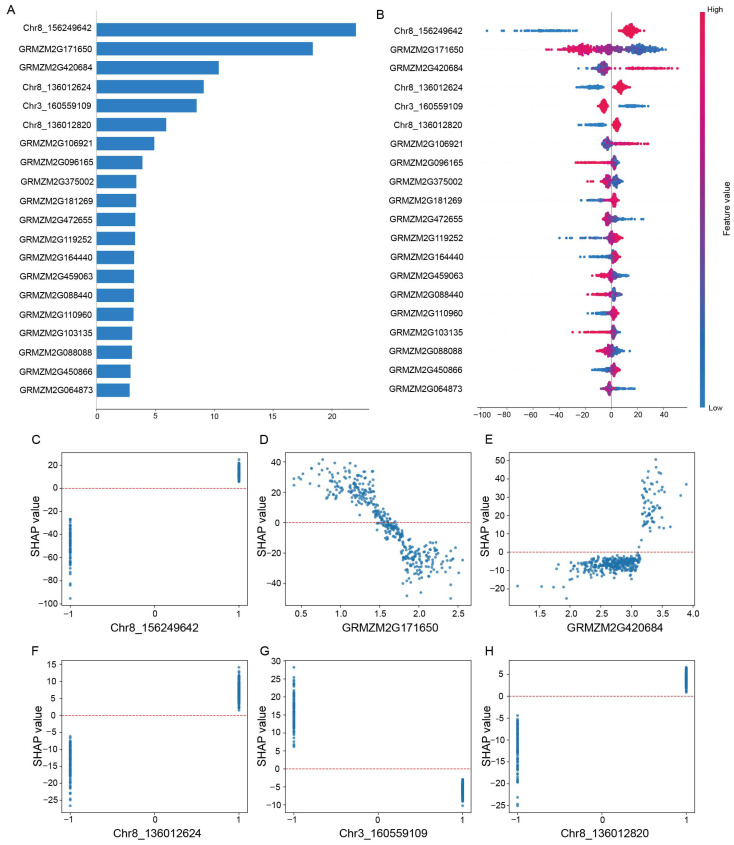
Feature importance and contribution to model output based on SHAP evaluation. (**A**) The top 20 features selected by the RF model, ranked by their SHAP importance scores. The *x*-axis represents the mean absolute SHAP value. (**B**) SHAP beeswarm plot illustrating how feature values influence the RF predictions. Red points indicate higher feature values/major allele (1), while blue points indicate lower feature values/minor allele (−1); positive and negative SHAP values reflect the direction and magnitude of each feature’s contribution to the model output. (**C**–**H**) Main effect SHAP dependence plots of the TOP 6 features. This chart characterizes the impact of different feature on maize flowering time under different genotypes and expression levels. Aggregation above the baseline at zero indicates that this genotype delays flowering time, while aggregation below baseline indicates an accelerated flowering time.

The feasibility of interpreting phenotypic contributions through SHAP values has been demonstrated [22]. Previous results indicated that Boruta feature selection can significantly reduce the number of features. Consequently, we first constructed the prediction models using the transcriptome and SNP data variables retained by Boruta, and SHAP values were subsequently used to rank the importance of each feature (Figure 5A). The beeswarm plot visualizes the specific influence of each feature value on the RF prediction output (Figure 5B). The top ranked SNP locus is Chr8_156249642 (GRMZM2G085438), the point color for this SNP reflects the minor allele (−1) in blue and the major allele (1) in red. The corresponding SHAP values represent the direction of the gene’s influence: positive SHAP values suggest that tendency to delay flowering, whereas negative values promote earlier flowering (Figure 5B).

From a vertical comparison perspective, Chr8_156249642, GRMZM2G171650, GRMZM2G420684, Chr8_136012624, Chr3_160559109, and Chr8_136012820 had the strongest effects on predicting maize flowering time. In addition, SHAP dependence plots are commonly applied to interpret the relationships between significant features and phenotypes [23]. For the six most impactful features, we further constructed SHAP dependency plots (Figure 5C–H). Notably, in Figure 5C,F,H, allele 1 of SNP exhibited aggregated SHAP values above the baseline, indicating that this genotype tends to delay flowering time. In contrast, allele −1 showed aggregated SHAP values below zero, suggesting a suppressive effect, contributing to earlier flowering. Similarly, for GRMZM2G171650 in Figure 5D, higher expression levels promoted flowering, whereas lower expression levels were associated with delayed flowering. Interestingly, GRMZM2G171650 corresponds to *ZmMADS69*, a well-known transcription factor regulating maize flowering time. *ZmMADS69* acts as a flowering promoter, and its overexpression leads to accelerated flowering. Furthermore, Chr8_136012624 and Chr8_136012820 map to different positions within the same gene GRMZM2G700665 (*ZmRap2.7*). Previous studies reported that CRISPR/Cas9 knockout mutants of the *ZmRap2.7* gene flowered approximately 2.6 days earlier than the wild type [24], further supporting the biological reliability of our SHAP interpretations. Thus, Chr8_156249642 (GRMZM2G085438) and GRMZM2G420684 also emerge as strong novel candidate genes underlying flowering time variation.

Taken together, by combining Boruta feature selection with SHAP-based interpretability, our machine learning framework successfully identified both known flowering time regulators and novel candidate genes. These findings confirm the biological interpretability of the model and provide important clues for further elucidating the genetic regulatory network underlying flowering time.

## 3. Discussion

### 3.1. Molecular Basis and Key Regulators of Flowering Time in Maize

Flowering time is regarded as a key agronomic trait that determines crop adaptation and yield, and its genetic architecture is known to be highly complex. It is influenced by photoperiod, circadian rhythms, endogenous hormonal signals, and multiple developmental signals. The elucidation of the flowering time regulation network plays an important role in advancing our understanding of plant developmental mechanisms, and it also provides valuable genetic resources for improving crop adaptability in different environments. In recent years, numerous genes involved in photoperiod responsiveness and circadian regulation have been identified. For example, it was previously shown that *ZmELF3.1* and *ZmELF3.2* are incorporated into protein complexes with *ZmELF4.1/4.2* and *ZmLUX1/2* as part of the maize circadian system. When the activity of these components was impaired, substantial delays in flowering were observed under both long-day and short-day conditions [25]. These findings indicate that circadian-clock elements play a pivotal role in regulating flowering time in maize. Notably, the 388-line panel used in this study spans diverse breeding backgrounds and environmental origins, including NSS, Flint, Popcorn, SSS, Iodent, and tropical germplasm, which likely experienced distinct photoperiod regimes during development [26]. This diversity provides an ecological and evolutionary context for our findings and highlights the relevance of clock-related genes identified in this study to environmental adaptation across diverse agro-ecological settings.

It is worth emphasizing that *ZmMADS69* and *ZmRap2.7* were identified in this study as key features most strongly associated with flowering time, and both genes have been independently validated in previous functional analyses (Figure 5). According to previous research, the MADS-box transcription factor *ZmMADS69* has been characterized as a major promotive regulator of flowering time that operates through the *ZmRap2.7–ZCN8* pathway. Rather than acting directly on the florigen gene, *ZmMADS69* functions upstream of the flowering repressor *ZmRap2.7*, suppressing its expression and consequently releasing the inhibition imposed on *ZCN8*. This regulation ultimately facilitates the floral transition [24].

In addition to confirmed flowering time genes, two candidate genes, GRMZM2G085438 (Chr8_156249642) and GRMZM2G420684, were also identified in this study (Figure 5). The maize gene GRMZM2G085438 is homologous to *Arabidopsis thaliana AT1G77420*, which encodes a member of the MAGL (monoacylglycerol lipase) family. MAGL proteins have been reported to participate in lipid catabolism, energy mobilization, and stress responses [27,28]. As lipid metabolism is closely linked to energy status, oxidized lipid-derived signaling, and the coordination of nutrient availability with developmental transitions [29], we speculate that changes in the expression or activity of GRMZM2G085438 could modulate lipid-mediated signaling or metabolic homeostasis in maize. This regulation may in turn influence the timing of floral induction, suggesting a potential role for this gene in the regulation of flowering time in maize. GRMZM2G420684 encodes a COI (Coronatine Insensitive) F-box protein, which has been shown to mediate the degradation of DELLA proteins, key repressors in the gibberellin (GA) signaling pathway [30]. In *coi* maize mutants, DELLA accumulation is associated with shortened internodes, delayed development, and reduced photosynthetic performance, suggesting that *COI* may influence the balance between growth and developmental progression through JA–GA crosstalk and DELLA turnover. These results suggest that *COI* may also function in regulating flowering time. Ultimately, functional validation of these candidate genes, for example by CRISPR-based gene editing and overexpression, is required to confirm the regulatory role of these genes in flowering time control in maize.

### 3.2. The Potential and Challenges of Machine Learning in Plant Genomic Prediction

Genomic prediction has been widely applied in both animal and plant breeding and continues to evolve alongside advances in computational methodologies. At present, no single algorithm universally performs best across all species, traits, and data structures. This limitation becomes particularly evident with the rapid expansion of high dimensional omics data, where traditional linear models struggle to capture the complex, nonlinear relationships among genotypes, phenotypes, and environments. In contrast, machine learning methods, with their stronger representational capacity, have emerged as a promising direction in genomic prediction research [31]. Although studies in livestock and aquaculture have demonstrated the predictive advantages of machine learning over classical linear models, systematic evaluations in plant breeding remain limited [32]. By comparing multiple models, including rrBLUP, RF, SVMrbf, SVMpoly, LightGBM, and XGBoost, this study further elucidates the applicability and limitations of machine learning in plant genomic prediction, providing insights for its future adoption in crop improvement.

With respect to model stability, RF and LightGBM showed consistently stable performance across various feature selection methods, whereas rrBLUP and SVMpoly were more sensitive, with large performance fluctuations (Figure 3). This robustness of RF may stem from its ensemble nature and random selection of features during tree construction, which makes it less dependent on specific feature subsets.

### 3.3. The Critical Role of Feature Selection in High Dimensional Omics Prediction

Previous studies have emphasized that feature selection is an effective strategy for addressing the “high dimensional, low sample size” challenge. It not only reduces the number of features and computational burden but also mitigates overfitting and improves prediction generalization ability [16,33]. In data structures where the number of predictors far exceeds the number of training samples, appropriately reducing dimensionality can effectively alleviate the “curse of dimensionality”. Even when feature selected models achieve prediction accuracy comparable to full feature models, the strategy still offers practical benefits, such as guiding the development of low density SNP arrays and reducing genotyping costs, thereby enhancing the economic feasibility of genomic selection in breeding programs [34]. In this study, Lasso and ElasticNet not only effectively reduced dimensionality but also maintained competitive prediction accuracy across different omics inputs. Starting from an initial set of approximately 332,177 markers, Lasso and ElasticNet reduced the feature space to an average of 595–6792 markers, while achieving mean PCC values of 0.65–0.66, which are comparable to those obtained using the full marker set with rrBLUP. Boruta further demonstrated a strong capacity for dimensionality reduction under high-dimensional settings (Figure 4 and Figure S3), retaining on average 54 informative markers, corresponding to a reduction of approximately 99.98% relative to the full feature set. From a practical perspective, reductions in marker density have direct implications for genotyping costs. When marker density is reduced to half of the original set, genotyping costs are expected to decrease to approximately two thirds of the original costs, whereas reducing marker density to one quarter of the original set results in estimated costs of about one half of the original [35]. Therefore, the substantial marker reductions achieved by Lasso, ElasticNet, and Boruta could translate into approximately 33–50% reductions in genotyping costs, with minimal loss in prediction accuracy. Collectively, these results demonstrate that appropriate feature selection enables a favorable trade-off between predictive performance and genotyping costs, facilitating the development of economically efficient and practically applicable low-density genomic prediction pipelines.

### 3.4. Limited Predictive Performance of Transcriptome-Based Models

Our study also revealed that prediction accuracy based on transcriptomic data was generally lower than that obtained with SNP data and showed reduced stability across different models (Figure 4). A possible explanation for this result is the developmental-stage mismatch between the transcriptomic data and the target trait. In this study, RNA-seq data were obtained from whole-seedling tissues at the V1 developmental stage, including both shoot and root tissues. Although flowering time is phenotypically expressed at later developmental stages, its genetic regulation is initiated much earlier during plant development. Transcriptomic profiles at the seedling stage can therefore capture genotype-dependent baseline transcriptional states, including genetic effects on gene expression, developmental potential, and early signaling pathways, which may be progressively amplified during subsequent development and ultimately influence flowering time. However, flowering time is governed by complex and dynamic developmental programs and the gene expression profiles at this early stage may not adequately capture the regulatory networks directly controlling flowering time, thereby limiting their predictive power. In addition to this developmental-stage distance, transcriptomic variation is highly sensitive to environmental conditions, leading to pronounced gene-by-environment (G × E) interactions that further reduce the robustness of transcriptome-based predictions. Together, these factors likely contribute to the limited predictive performance observed for transcriptomic models. Future studies could address these limitations by incorporating transcriptomic data from developmentally relevant stages, or by integrating longitudinal expression profiles with environmental covariates. Such approaches, combined with modeling frameworks that explicitly account for G × E interactions and nonlinear regulatory relationships, may substantially enhance the predictive utility of transcriptomic data for complex trait prediction and functional gene analysis.

### 3.5. Model Interpretability and Gene Mining Enhanced Through SHAP

SHAP values have been widely applied in various research fields, but their application in crop genetics remains relatively limited. In recent studies, the application of SHAP values has been explored in several crops. In maize, within the Cropformer framework, SHAP values derived from XGBoost were employed to prioritize genomic loci that may influence maize tasseling time. This approach highlighted high-ranking loci associated with functional variants, demonstrating the utility of SHAP in linking model-derived feature importance with biologically relevant candidate regions [36]. In rice, SHAP values were used to predict genes contributing to drought tolerance, identifying *OsRAV11* as a potential candidate. Functional validation experiments suggested *OsRAV11* plays broader roles in both drought response and flowering regulation [37]. Similarly, DPCformer utilized SHAP values to identify key SNPs affecting plant height and ear weight, further confirming the practicality of SHAP in gene discovery for complex agronomic traits [38].

In this study, we systematically evaluated multiple feature selection strategies and prediction models to extract stable and biologically meaningful features from multi-omics data, and further interpreted the prediction models using SHAP in a RF framework. SHAP provides not only global assessments of feature importance but also quantifies local contributions at the sample level, enabling the identification of core regulatory factors within complex models. In addition, the RF + SHAP framework offers transparent and interpretable feature-level attributions: SHAP values are additive and based on Shapley theory, ensuring that the contribution of each feature to individual predictions is explicit and stable. Compared with interpretability methods for deep-learning models, RF + SHAP is computationally efficient, requiring only standard CPU resources, and exhibits high reproducibility, as feature attributions remain consistent across runs with fixed data and random seeds. This combination of transparency, efficiency, and reproducibility provides a robust approach for extracting biologically meaningful insights and informing downstream experimental validation. Through this approach, we successfully captured known key genes, including *MADS69*, validating the effectiveness of SHAP in both model interpretation and gene discovery (Figure 5). Nevertheless, numerous yet unknown genes remain to be discovered. In the future, integrating more adaptive feature selection strategies, such as automated feature selection with deep neural networks or reinforcement learning–based optimization, along with functional validation experiments like qPCR or CRISPR, may further enhance the accuracy and biological reliability of candidate gene identification. Such approaches are expected to provide more precise targets for understanding flowering time regulation and advancing crop genetic improvement.

It should also be noted that SHAP values have inherent limitations. In high-dimensional omics data, strong correlations among features are common due to linkage disequilibrium or gene co-expression, which may lead to instability in SHAP-based feature attributions, with importance being redistributed among highly correlated predictors. Therefore, SHAP results should be interpreted with caution when identifying candidate genes and drawing biological conclusions. Nevertheless, several strategies can help alleviate this issue, including grouping highly correlated features, evaluating the robustness of SHAP attributions across different cross-validation folds or resampled datasets, and prioritizing candidate genes that consistently exhibit stable contributions across data partitions. Incorporating such robustness-oriented analyses in future studies may further enhance the reliability of SHAP-based interpretations.

## 4. Materials and Methods

### 4.1. Plant Materials

A total of 388 maize inbred lines were selected from a panel of 503 maize pan-genome lines, for which data on flowering time [26] genotype, and transcriptome [39] were available. Maize plants were cultivated under controlled greenhouse conditions with a 16 h light/8 h dark photoperiod and day/night temperatures of 27 °C/24 °C. Each pot (30 cm top diameter, 28 cm height; 14.5 L volume) contained six plants grown in Metro-Mix 300 substrate (Sun Gro Horticulture) without additional fertilization. Whole-seedling samples, including both shoot and root tissues, were collected at the V1 developmental stage for transcriptomic profiling. The data processing procedures followed the methodology described by Azodi et al. [40]. The final genomic dataset contained 332,177 high-quality SNP markers, after the removal of unmapped loci and markers with low minor allele frequency. SNP markers were encoded using a −1/0/1 scheme, corresponding to genotypes aa, Aa, and AA, respectively, where the more frequent (major) allele (AA) was coded as 1. The transcriptomic dataset consisted of 31,237 expressed genes, following filtering steps that excluded genes with missing coordinates or near-zero variance across lines. Expression values were provided as log-transformed TPM and used directly for downstream analyses.

### 4.2. Methods

**Figure 6:**
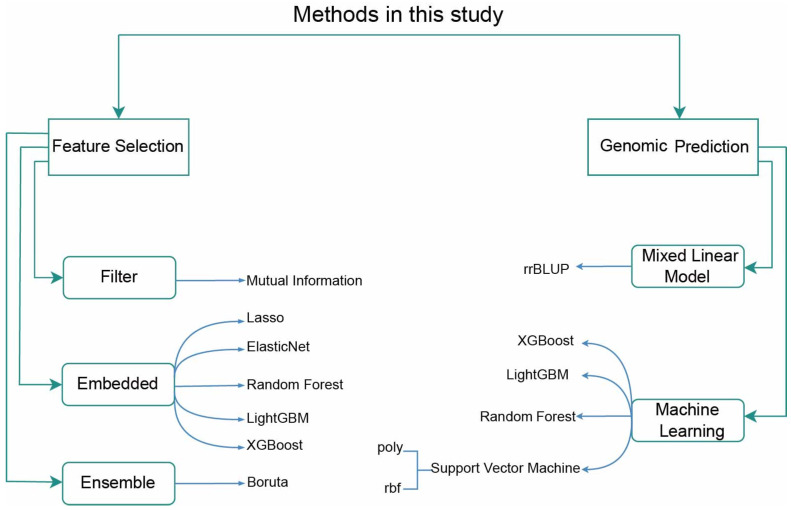
An overview of the methods used in this study.

An overview of the methods used in this study is shown in Figure 6.

### 4.3. Feature Selection Methods

A ten-fold cross-validation scheme was applied to the entire dataset to assess model performance. Specifically, the full dataset was randomly partitioned into ten approximately equal-sized folds. In each iteration, one fold was held out as the test set, while the remaining nine folds were used for feature selection and model training optimization. Model performance was evaluated on the held-out fold in each iteration, and the final prediction accuracy was reported as the average across all ten folds. In each iteration of model performance assessment (outer loop), feature selection was performed exclusively within the training set with a nine-fold cross-validation (inner loop) (Figure S1), thereby preventing information leakage and ensuring an unbiased estimation of model generalization performance. In tree-based models such as LightGBM and XGBoost, feature importance values are computed differently and often produce extremely small nonzero values (e.g., 1 × 10^−17^). To avoid retaining features that are essentially irrelevant due to numerical noise, we applied a threshold of 1 × 10^−5^ for these models. For other models, features with importance greater than zero were retained.

#### 4.3.1. Feature Selection: Mutual Information

In probability theory and information theory, the mutual information between two random variables measures their degree of interdependence. Specifically, mutual information quantifies the reduction in “information” of one random variable when the other is known. Mutual information is closely related to entropy, which is a fundamental concept in information theory that quantifies the “information content” within a random variable. It is widely used in the field of computer science [41] and has also been applied in genomic prediction by previous studies [42]. In this study, the mutual_info_regression method was used to calculate the mutual information score between each feature and the target phenotype. We retained features with mutual information scores greater than zero, considering these features to have a positive contribution to the target phenotype and potentially contain key biological information. The loss function is defined as follows:

$$I\left(X;Y\right)=\iint p\left(x,y\right)\log\left(\frac{p\left(x,y\right)}{p\left(x\right)p\left(y\right)}\right)dx\;dy$$

where *I*(*X*; *Y*) denotes the mutual information between the features *X* and the target representation *Y*. *p*(*x*, *y*) denotes the joint distribution of *X* and *Y*, and *p*(*x*) and *p*(*y*) denote their respective marginal distributions.

#### 4.3.2. Feature Selection: Lasso

Lasso (Least Absolute Shrinkage and Selection Operator) regression is a linear modeling approach that integrates variable selection and regularization, making it particularly valuable in high-dimensional data analysis. By applying L1 regularization to the regression coefficients, Lasso effectively shrinks the coefficients of irrelevant features to zero, thereby highlighting the most significant variables. This characteristic not only enhances the interpretability of the model but also reduces the risk of overfitting. This method has been widely applied in fields such as economics and finance to improve predictive performance and uncover important variables that might be overlooked by traditional approaches [43,44]. In this study, feature selection was performed with the Lasso regression model implemented in the ‘sklearn.linear_model.Lasso’ class. To ensure model convergence, we trained for a maximum of 10,000 iterations. After model fitting, the absolute values of the regression coefficients were used as feature importance scores. Features with importance values greater than zero were retained because they were considered informative for prediction. Lasso’s loss function is defined as follows:

$$\underset{w}{\min}\left.\left\{\;||y\;-\;Xw|{|}_{2}^{2}+\;\alpha ||w|{|}_{1}\right\}\right.$$

where *y* denotes the target representation vector, *X* denotes the genotype feature matrix, *w* denotes the regression coefficients, and *α* is a regularization parameter selected via cross-validation.

#### 4.3.3. Feature Selection: ElasticNet

ElasticNet is a widely adopted regularization regression method that combines the strengths of Lasso (L1 regularization) and Ridge (L2 regularization), making it suitable for high-dimensional data and feature selection. It is particularly effective when there is multicollinearity between features or when the number of features far exceeds the number of samples. ElasticNet controls model complexity through a penalty term, preventing overfitting while retaining the important characteristics of variable selection and model interpretability. ElasticNet has been extensively used in cancer prognosis [45]. The absolute values of the regression coefficients obtained from the fitted model are used as feature importance scores. Features with importance values greater than zero were retained, as they were considered to contribute meaningfully to the model. To ensure numerical stability and convergence, the maximum number of iterations (max_iter) was set to 10,000, which helped the optimization process converge reliably and ensured reproducible feature selection results. The loss function of ElasticNet can be expressed as follows:

$$\underset{w}{\min}\left\{\left.\;||y\;-\;Xw{\left|\right|}_{2}^{2}+\;\alpha \left.\left(\;\left(1\;-\;L1\_ratio\right)||w|{|}_{2}^{2}+\;L1\_ratio\;||w|{|}_{1}\right.\right)\right.\right\}$$

where *y* denotes the target representation vector, *X* denotes the genotype feature matrix, and *w* denotes the regression coefficients.

#### 4.3.4. Feature Selection: RF

RF, also known as a Random Decision Forest, is an ensemble learning method widely used for classification, regression, and other tasks. It operates by constructing a large number of decision trees during training. One of the key advantages of RF is its ability to mitigate the tendency of individual decision trees to overfit the training data [46]. In recent years, RF has been increasingly applied to human genomic data analysis [47]. RF was used as a feature selection tool by leveraging the importance scores produced by the ensemble. A RandomForestRegressor with 100 trees was fit solely for the purpose of estimating feature importance, which was computed based on each feature’s contribution to impurity reduction across tree splits. Features with an importance value greater than zero were retained. This RF model was used exclusively for importance estimation rather than for phenotype prediction.

#### 4.3.5. Feature Selection: LightGBM

LightGBM (Light Gradient Boosting Machine) is a highly efficient, distributed machine learning algorithm based on the gradient boosting framework. It has gained widespread application due to its fast computation speed, low memory consumption, and strong capability in handling large-scale data. Feature selection with LightGBM was conducted using the feature importance values generated from the trained gradient boosting framework. A LGBMRegressor with 100 estimators was trained on the dataset, and feature importance was quantified based on each feature’s contribution to the split gain in the boosting process. Features with importance scores exceeding 1 × 10^−5^ were retained. This LightGBM model was used solely for importance estimation rather than for downstream prediction.

#### 4.3.6. Feature Selection: XGBoost

XGBoost has been proven to be an effective, feasible, and relatively mature ensemble gradient boosting machine learning algorithm. However, it may consume a large amount of memory and perform poorly on unstructured or sparse data [48]. Feature selection XGBoost was performed based on the feature importance scores derived from the trained gradient boosting model. An XGBRegressor with 100 estimators was fitted to the training data, and the importance of each feature was computed according to its contribution to the model’s split decisions. Features with importance scores greater than 1 × 10^−5^ were retained. This XGBoost model was used exclusively for estimating feature importance and not for phenotype prediction.

#### 4.3.7. Feature Selection: Boruta

Boruta is a feature selection algorithm based on the Random Forest (RF) method. It evaluates feature importance by using random controls (adding random noise features as a comparison) to identify stable and important features, demonstrating high robustness. If the importance of a real feature is significantly higher than that of the shadow features, it is considered to make a stable contribution to the target variable [49]. Boruta eliminates unimportant features through an iterative process, with a maximum of 100 iterations (max_iter = 100), to determine the final set of retained features. The feature selection of Boruta was implemented by the library of boruta (version 0.4.3) in Python.

### 4.4. Machine Learning Algorithm Models and Traditional Linear Model

This study utilized five machine learning algorithms: RF, Support Vector Machine with Radial Basis Function kernel (SVMrbf) and Polynomial kernel (SVMpoly), and LightGBM and XGBoost. Hyperparameter tuning was conducted using a grid search strategy with carefully predefined parameter ranges, nested within the model performance assessment to avoid optimistic bias. The number of estimators (n_estimators) was fixed at 100 and was therefore excluded from the hyperparameter search. A fixed random seed (random_seed = 42) was used for the consistent partitioning of samples into training, validation, and test sets. An external 10-fold cross-validation was performed for model evaluation (outer loop) and a nine-fold cross-validation for feature selection (inner loop) within the training set (Figure S1). We repeated the 10-fold cross-validation 5 times, totaling 50 (10 × 5) iterations for each model performance assessment. Each iteration, the Pearson correlation coefficient (PCC) between the predicted and actual trait values in the test set was calculated to assess the final model’s performance. Model fitting for rrBLUP was carried out through the mixed.solve procedure available in the R (version 4.5.1) of the rrBLUP package (version 4.6.1). The model was fitted as:

y = Xβ + Zu + ε

where β is a vector of fixed effects and u is a vector of random marker effects, X the design matrix (n × *p*) for the fixed effects, in which a vector of ones was used to model the intercept. Z is the marker incidence matrix, and u represents the additive effects associated with individual markers. The residual term ε captures unexplained variation.

RF was implemented using a RandomForestRegressor with 100 trees, enabling nonlinear relationship modeling through ensemble averaging. Parameters tested included max tree depth (3, 5 and 10) and max_features (0.1, 0.25, 0.5, 0.75, “sqrt”, “log2”, “None”). Gradient boosting models were implemented using XGBRegressor for XGBoost and LGBMRegressor for LightGBM. For XGBoost, max_depth was tuned over values 3, 5, and 10, while for LightGBM, max_depth (3, 5, 10) and num_leaves (31, 63) were optimized. To complement these tree-based learners, two support vector regression models were included. For SVMrbf, hyperparameter tuning was performed over the regularization parameter (C = 0.001, 0.01, 0.1, 0.5, 1, 10, 50) and the kernel width parameter (gamma = 10^−5^ to 10^1^, logarithmically spaced). For SVMpoly, hyperparameters included the regularization parameter (C = 0.001, 0.01, 0.1, 0.5, 1, 10, 50), the polynomial degree (degree = 2, 3, 4), and the kernel coefficient (gamma = 10^−5^ to 10^1^, logarithmically spaced). These five models collectively provide a diverse set of learning strategies covering ensemble methods, gradient boosting, and kernel-based nonlinear regression. All analyses were conducted in Python 3.9.18, using the following key libraries: xgboost (version 2.1.4), lightgbm (version 4.6.0), scikit-learn (version 1.6.1), numpy (version 1.26.4), and pandas (version 2.3.0).

### 4.5. Interpretation of Candidate Genes via Explainable Artificial Intelligence (SHAP)

To elucidate the decision-making mechanism of the RF model and quantify the contribution of each feature, SHAP (SHapley Additive exPlanations) analysis was performed using TreeExplainer based on the trained models. SHAP values measure the influence of individual features on model output, capturing both the main effects of gene expression or SNPs and potential interactions among features. After model training, SHAP values were calculated for all input features and ranked by their mean absolute contributions. The top 20 features were then selected to identify the most influential molecular determinants associated with flowering time prediction.

All SHAP computations and visualizations were conducted in a Python 3.9.18 environment using the pandas, scikit-learn, shap (version 0.49.1), and matplotlib (version 3.9.4) libraries. As a well-established interpretability framework, SHAP has been widely validated in biological prediction models, with its effectiveness demonstrated in multiple studies [18,36,37].

## 5. Conclusions

This study systematically benchmarked the predictive performance of 42 FS–GP combinations, consisting of 7 feature selection methods and 6 genomic prediction models, for flowering time prediction. The results showed that linear methods such as Lasso and ElasticNet, as well as nonlinear methods like Boruta, are particularly effective in dimensionality reduction, significantly reducing the number of features while retaining biologically meaningful signals. In terms of prediction models, random forest performed most stably and accurately when integrating SNP and transcriptome data. The SHAP-based interpretation of the random forest model identified known flowering time regulatory genes, including *ZmMADS69* and *ZmRap2.7*, and further revealed additional candidate genes potentially involved in the flowering regulatory network. Overall, these findings provide practical guidance for selecting effective analytical strategies and contribute to a deeper understanding of the genetic basis of flowering time. For practical applications in breeding programs, we suggest considering an ElasticNet/Lasso/Boruta → Random Forest → SHAP workflow, which aims to balance predictive accuracy with interpretability. A concise best-practice checklist can further facilitate implementation: (1) perform hyperparameter tuning on the training data to determine optimal model parameters; (2) conduct feature selection and model evaluation using 10-fold cross-validation; and (3) use SHAP values to interpret feature contributions and identify key candidate genes.

## Data Availability

All relevant data are within the paper and its Supplementary Materials. The scripts are available in the release package on github (https://github.com/Jnhcau/FS-GP, accessed on 6 February 2026).

## Supplementary Materials

The following supporting information can be downloaded at: https://www.mdpi.com/article/10.3390/ijms27041635/s1.
