# Supplementary figures and images for: Benchmarking Feature Selection Methods and Prediction Models for Flowering Time Prediction in Maize
Source: Int J Mol Sci. 2026 Feb 7;27(4):1635. doi: 10.3390/ijms27041635 (PMC12941171; doi:10.3390/ijms27041635)

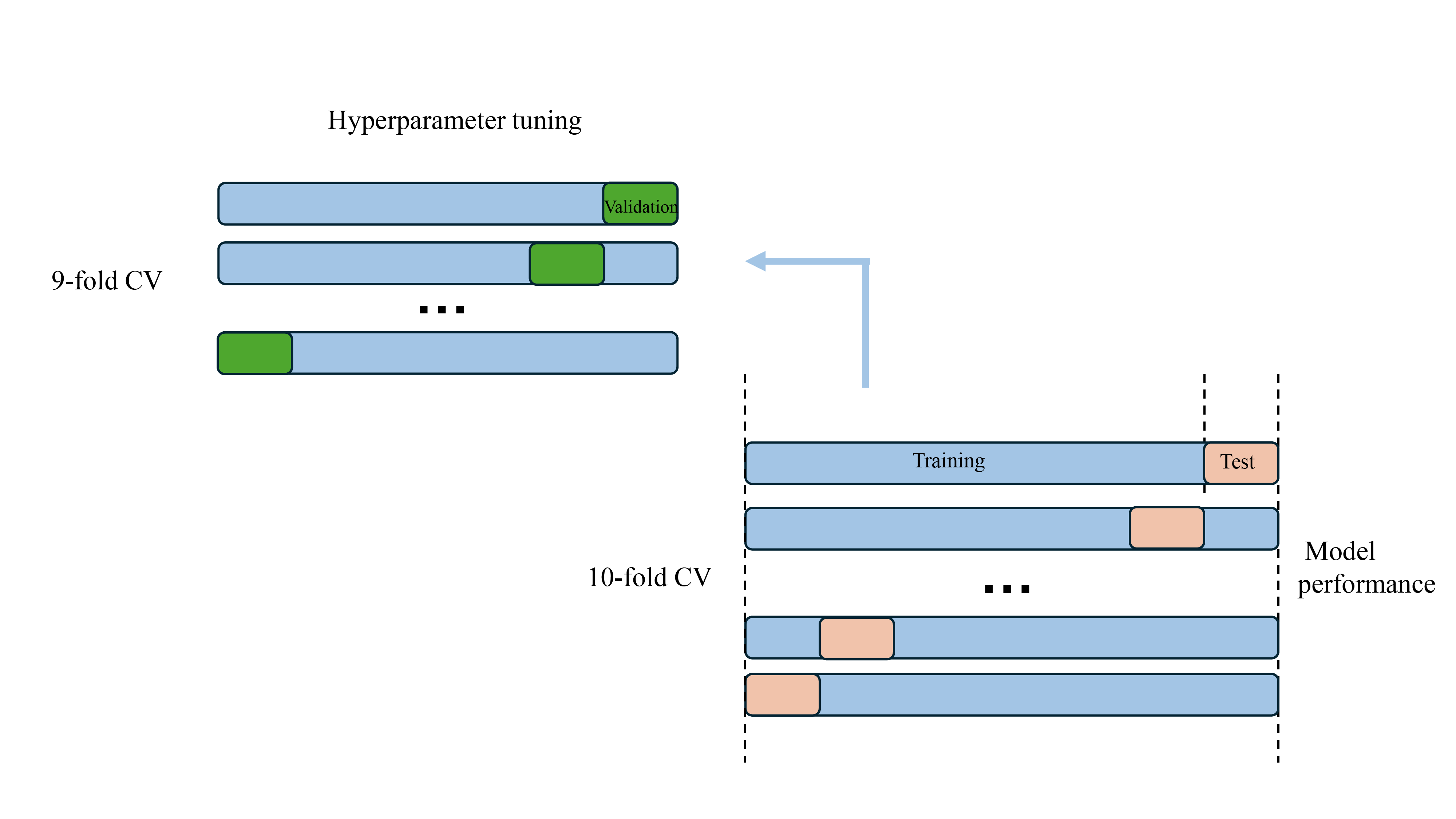

Supplement: Supplementary file 1 [file ijms-27-01635-s001.zip › Figure.S1.Model performance and hyperparameter tuning framework.tif]

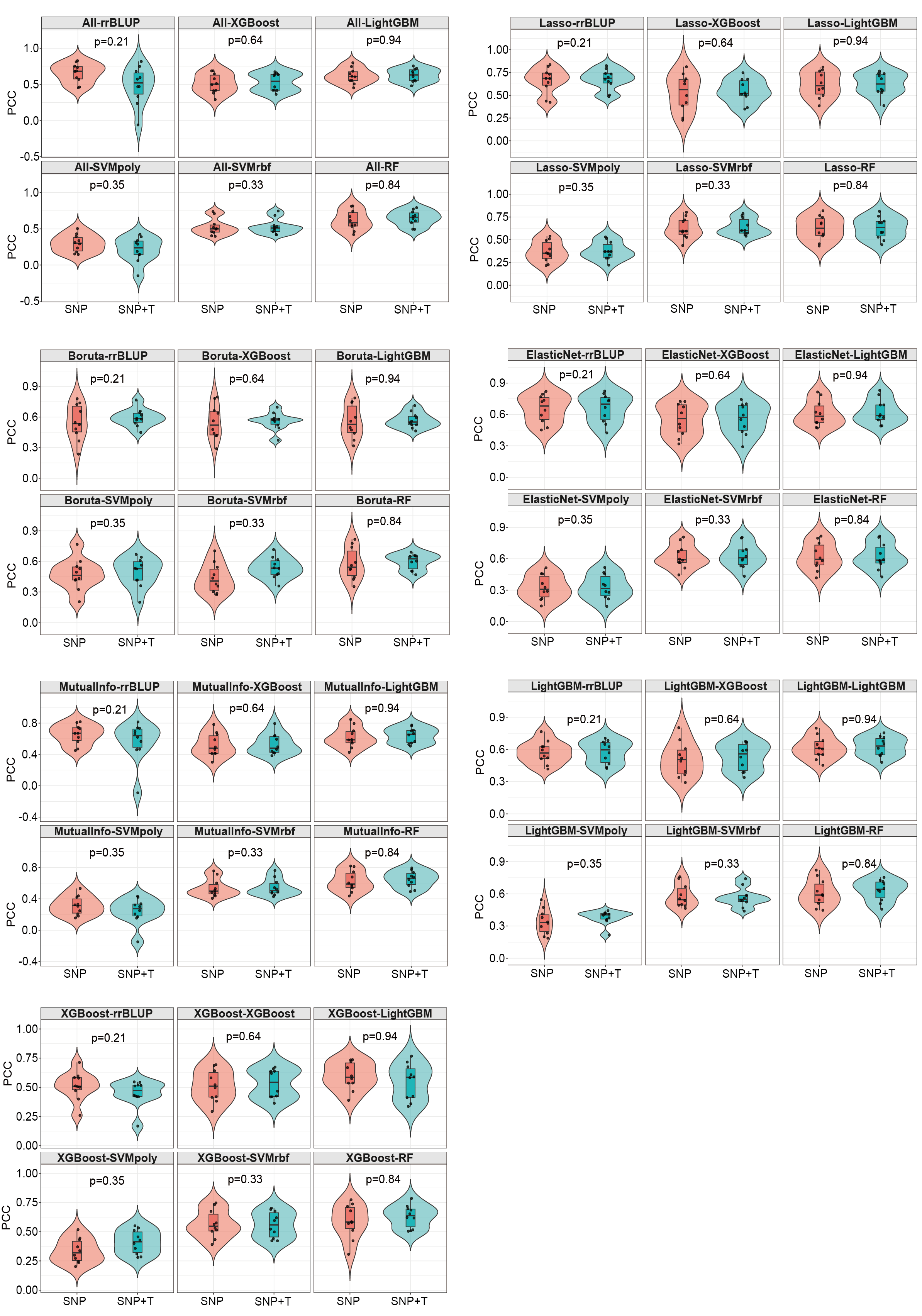

Supplement: Supplementary file 1 [file ijms-27-01635-s001.zip › Figure.S2.Distribution of PCC across cross-validation folds for SNP and SNP + Transcriptome models.tif]

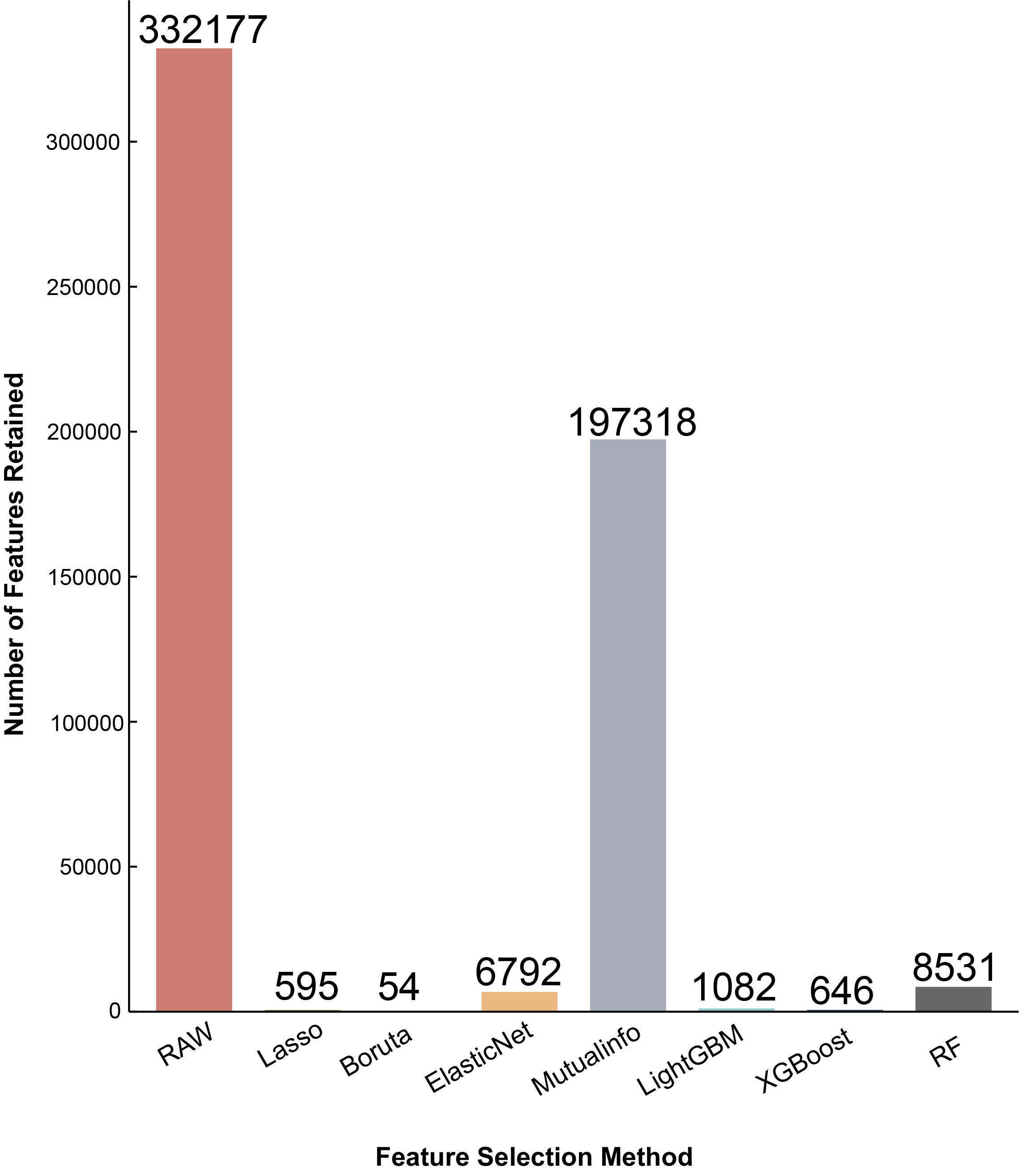

Supplement: Supplementary file 1 [file ijms-27-01635-s001.zip › Figure.S3.The average number of features retained by each feature selection method (SNP).tif]
